# 

**Published:** 1888-01

**Authors:** 


THE COLON OF THE HORSE—Its Diseases and Derangements. Thomas Walley, M.R.C.V.S.... 1
SOME RARE FORMS OF EYE DISEASE IN VETERINARY PRACTICE—Illustrated. W. R. Burke, V.S., A.D.V.. .......... 18
THE HISTOLOGY AND PATHOLOGY OF REPRODUCTION. Henry O. Marcy, A.M., M.D., LL.D. 19
SOME NOTES UPON SOFT-SHELLED TURTLES, AND THE ANATOMICAL VAGARIES OF ASPIDONECTES SPINIFER. G. Archie Stockwell, M.D., F.Z.S.......... 28
COMPARATIVE PSYCHOLOGY. T. Wesley Mills, M.A., M.D...... 43
LYSSA AND THE PASTEUR FIASCO. N. E. Brill, A.M., M.D........ 55
THE MONTREAL VETERINARY COLLEGE, AND ITS FOUNDER AND PRINCIPAL—Illustrated........ 78
EDITORIAL DEPARTMENT........ 85
SELECTIONS FROM FOREIGN JOURNALS........ 100
PROCEEDINGS OF VETERINARY MEDICAL SOCIETIES........ 105
REVIEWS........ 109

